# Splenic Lymphangioma Mimicking Lymphomatous Involvement: A Case Report with Review of the Literature

**DOI:** 10.1155/2023/9969213

**Published:** 2023-06-20

**Authors:** Neda Soleimani, Fatemeh Pouraminaee, Mohammad Hossein Anbardar, Ali Bahador, Benyamin Rahimi, Sahand Mohammadzadeh, Fatemeh Aghakhaninejad, Mohammad Farahmand, Mahsa Hasani

**Affiliations:** ^1^Department of Pathology, Shiraz Medical School, Shiraz University of Medical Sciences, Shiraz, Iran; ^2^Shiraz Transplant Center, Abu Ali Sina Hospital, Shiraz University of Medical Sciences, Shiraz, Iran; ^3^Pathology Research Center, Shiraz University of Medical Sciences, Shiraz, Iran

## Abstract

Lymphangioma is a benign malformation of lymphatic vessels usually found in the head and neck areas or axilla. They may involve visceral organs with a lower percentage. Splenic lymphangioma is a rare tumor. This disease is often seen in children but may be diagnosed incidentally in adults. Most patients are asymptomatic, but in large and multifocal lesions, the patient may have some nonspecific symptoms such as abdominal pain, abdominal distention, nausea, vomiting, and loss of appetite. Physical examination may show no specific findings or detect palpable masses. The preoperative diagnosis of splenic lymphangioma is challenging. Histopathological evaluation and sometimes immunohistochemistry tests can result in a definitive diagnosis. In this study, we present an 18-year-old man, with Burkitt's lymphoma who underwent laparotomy and total splenectomy as a result of cystic lesions discovered accidentally during imaging with the final diagnosis of splenic lymphangioma after histopathological evaluation.

## 1. Introduction

Lymphangioma is a benign neoplasm of lymphatic vessels, often affecting children's head and neck areas. It is uncommon to find lymphangiomas in abdominal organs such as the kidney, adrenal, small bowel mesentery, liver, and spleen. Splenic lymphangioma is very rare and is often found incidentally. Nevertheless, some patients may experience nonspecific signs and symptoms such as abdominal pain and distention. Preoperative diagnosis would be difficult, and overdiagnosis might happen because radiological findings are also not specific. The ultimate diagnosis is usually made by histopathological examination [1–7]. Herein, we report a case of splenic lymphangioma in an adult patient who had a previous case of Burkitt's lymphoma.

## 2. Case Report

**Figure 1:**
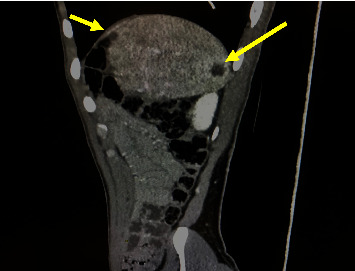
Abdominal spiral CT scan with contrast shows a few near together cystic lesions with faint enhancement after administration of contrast (arrows).

**Figure 2:**
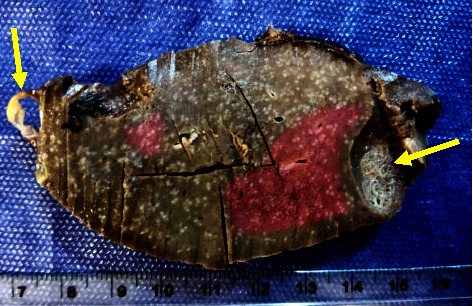
Cut section of the spleen shows two well-defined multiloculated cystic lesions (arrows).

**Figure 3:**
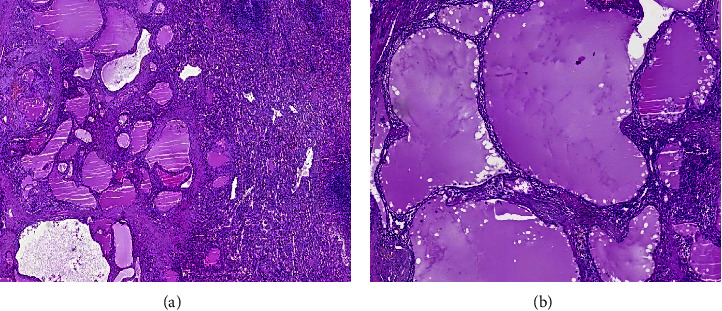
Microscopic assessment shows vascular spaces containing pinkish fluid (a) (*H*&*E* × 40). The spaces are lined by endothelial cells (b) (*H*&*E* × 200).

An 18-year-old man was transferred to our center to be evaluated for splenic lesions, which were found incidentally during a routine annual follow-up for Burkitt's lymphoma. He had no history of abdominal pain, fever, or weight loss. He has been a case of Burkitt's lymphoma of the stomach since seven years ago and has received chemotherapy for one year. There was no sign of a disease recurrence, and all earlier ultrasonographic evaluations were unremarkable until the last follow-up, which revealed splenic lesions. Other past medical and family histories were not significant. On physical examination, blood pressure was 110/70 mmHg with a normal pulse rate and temperature, 68 beats per minute and 37.1°C, respectively. The abdomen was soft, and there was no organomegaly. Complete blood count (CBC) (white blood cell (WBC): 4.9 × 103/*μ*l, hemoglobin: 14.2 g/dl, and platelet count: 211 × 103/*μ*l) and liver enzymes, including aspartate aminotransferase (AST), alanine aminotransferase (ALT), and alkaline phosphatase (ALP), were within normal ranges. Abdominopelvic ultrasonography revealed a hyperechoic mass measuring 24 mm and a cystic lesion measuring 9 mm in the spleen parenchyma. A spiral CT scan of the abdomen with contrast showed a few near together cystic lesions up to 18 mm in the anterosuperior aspect and a hypoattenuated subcapsular lesion measuring 24 mm in the posterior aspect of the spleen with faint enhancement after administration of contrast, suggestive of lymphomatous infiltration (Figure 1). The patient underwent laparotomy. The splenic lesions were multiple at the time of the operation, so a diagnostic splenectomy was performed for him. Gross sectioning of the spleen showed two multiloculated cystic lesions, the largest measuring 1.5 × 1 × 1 cm and containing some brownish fluid (Figure 2). Histological examination showed multiple large irregular cystic spaces lined by endothelial cells with smooth pinkish fluid in the lumen and lymphoid aggregation consisting of lymphangiomas (Figures 3(a) and 3(b)). Two months later, no clinical or radiologic deficits were noted in a follow-up visit. We obtained the patient's informed consent for publishing the case report and images. Our institutional approval was not required to publish the case details.

## 3. Discussion

Splenic lymphangioma is a benign cystic neoplasm initially described by Frink in 1885, accounting for <0.007% of all tumors. It typically affects pediatric patients but is rarely found in adults [6, 8].

Sequestration of lymph tissue, aberrant lymph vessels budding, lack of integration with the venous system, and obstruction of lymph vessels are some theories to explain the pathogenesis of lymphangiomas.

The failure of the primordial lymphatics to connect to the main lymphatic vessels or veins, the lymphatic vessels' inability to drain into the veins, the abnormal budding of the lymphatic vessels, and chronic inflammation are a few of the hypotheses that have been suggested for the etiopathogenesis of lymphangiomas. Vascular endothelial growth factor-C (VEGF-C) and vascular endothelial growth factor-*R*3 are also elevated. However, in adult patients, as in our case, only a tiny defect often exists in the lymphatic system, which can be compensated under healthy conditions. Pathologic conditions associated with an increased lymphatic volume may then result in the development of lymphangioma. There is speculation that trauma and infectious diseases are the catalysts for this development [6, 9, 10]. Similarly, the fact that our patient developed a new splenic lesion that was not present in previous radiologic studies suggests that prior surgery and chemotherapy may have served as a trigger.

Clinical presentations depend on the tumor size. Most patients are asymptomatic, and the tumor is found incidentally. Large and multifocal lesions can, however, cause nonspecific symptoms such as abdominal pain, abdominal distention, nausea, vomiting, and loss of appetite, which are primarily brought on by the compression of nearby organs. Post-traumatic and spontaneous rupture of splenic lymphangiomas has also been reported. Moreover, it can lead to coagulopathy, hemorrhage, and portal hypertension. Physical examination is unremarkable or may detect a palpable mass [1, 11–13].

Radiological findings are generally not specific. Splenic lymphangiomas in ultrasonography may appear as anechoic, hypoechoic, or hyperechoic lesions depending on whether the lymphatic fluid is infected, hemorrhagic, or hyperlipidemic. They have different diameters, with or without internal septation. Calcification is a nonspecific finding and may also be observed in splenic hydatid cysts. Like in our case, computed tomography (CT) scan reveals single or multiple well-defined thin-walled lesions that are usually hypodense, homogenous, and without significant contrast enhancement [2, 12, 14]. The lesions are often well-vascularized in angiography.

Magnetic resonance imaging (MRI) shows hypointense cystic lesions in T1-weighted images, yet on T2-weighted images, they look hyperintense when filled with hemorrhagic or proteinaceous material. There have been some reported instances of lymphangioma without F-18-fluorodeoxyglucose (FDG) uptake on positron emission tomography (PET) scans. However, they may exhibit slightly enhanced F-18 FDG uptake as a result of lymphocytes and fluid joining the dilated lymphatic vessels [1, 6, 15–19].

Gross appearance shows single or multiple cystic lesions in various sizes with a thick fibrous wall filled with clear fluid. Multiple lesions may have a honeycomb look. Histopathological evaluation reveals cystic lesions lined by endothelial cells and filled with an amorphous eosinophilic proteinaceous substance [2, 20, 21]. Moreover, lymphangiomas are divided into three categories based on the size of the dilated lymphatic channels: (1) capillary (supermicrocystic), (2) cavernous (microcystic), and (3) cystic (macrocystic) [6].

Immunohistochemistry can be used when there is a doubt about the ability to differentiate cystic lesions of splenic lymphangioma from other cystic lesions such as mesothelial cysts, epidermoid cysts, and parasite cysts. The cells lining the cystic walls in splenic lymphangioma stain positive for CD34, CD31, factor VIII, vascular endothelial growth factor 3, and podoplanin [22–24].

Total splenectomy is the recommended treatment choice after the diagnosis is confirmed to reduce the risk of consequences such as infection, bleeding, growth, and expansion of the lesions and spleen rupture. Splenic lymphangioma has a favorable outcome after total splenectomy, and the possibility of malignancy is scarce [1, 3, 5].

Our patient's history of Burkitt's lymphoma resulted in a misdiagnosis of lymphomatous involvement in a CT scan study. However, if used, MRI and PET scans could aid with a more precise diagnosis and prevent misdiagnosis. In addition, since the lesions were located superficially in the spleen, a more accurate preoperative diagnosis might result in cyst excision rather than an unnecessary total splenectomy.

## 4. Conclusion

Splenic lymphangioma is an extremely rare tumor. Although this benign entity frequently affects children, it should be taken into consideration as a differential diagnosis in adults with acquired cystic lesions, especially in those who have already experienced intra-abdominal stress (surgery, trauma, infection, and so on).

## Data Availability

All data generated or analyzed during this study are included in this published article.

## Abbreviations

CBC: Complete blood count
WBC: White blood cell
AST: Aspartate aminotransferase
ALT: Alanine aminotransferase
ALP: Alkaline phosphatase
CT: Computed tomography
MRI: Magnetic resonance imaging
FDG: Fluorodeoxyglucose
PET: Positron emission tomography.
